# Development and validation of brief measures of workplace loneliness

**DOI:** 10.1093/joccuh/uiaf068

**Published:** 2025-11-27

**Authors:** Izumi Ayase, Akihito Shimazu, Masahito Tokita, Kentaro Sakamaki, Norito Kawakami

**Affiliations:** Graduate School of Media and Governance, Keio University, Shonan Fujisawa Campus, Kanagawa, Japan; Faculty of Policy Management, Keio University, Shonan Fujisawa Campus, Kanagawa, Japan; Keio Research Institute at SFC, Keio University, Shonan Fujisawa Campus, Kanagawa, Japan; Faculty of Health Data Science, Juntendo University, Hongo Campus, Bunkyo, Tokyo, Japan; Department of Mental Health, Graduate School of Medicine, The University of Tokyo, Bunkyo, Tokyo, Japan

**Keywords:** workplace loneliness, loneliness measurement, employee well-being

## Abstract

**Objectives:**

This study aimed to develop and validate 2 concise measures of workplace loneliness.

**Methods:**

A 3-item scale for loneliness at work (SLAW-3) and a single-item scale for loneliness at work (SLAW-1) were developed by modifying existing scales for general loneliness. A cross-sectional online survey was conducted of 1228 full-time employees in Japan to test the reliability and validity of these scales. Internal consistency of the SLAW-3 was assessed using Cronbach α coefficient, and the construct validity was examined through correlations with established measures of workplace loneliness, psychological distress, well-being, self-rated health, and workplace social support.

**Results:**

The SLAW-3 demonstrated strong internal consistency (Cronbach α = .91) and unidimensionality. It correlated positively with established measures of workplace loneliness and psychological distress measures and negatively with well-being and workplace social support measures. The SLAW-1 also demonstrated significant correlation with the SLAW-3 and with psychological indicators such as distress, well-being, self-rated health, and workplace social support.

**Conclusions:**

The SLAW-3 was found to be a reliable and valid measure of workplace loneliness. The SLAW-1 also demonstrated adequate construct validity, despite its single-item format. Both scales are short and may be effectively used to assess workplace loneliness in large-scale employee surveys or brief screenings.

## Teaser

**Key points**

**What is already known on this topic**
 

- Existing measures of workplace loneliness are often lengthy and not always practical for repeated or large-scale use.
- Previous research has established that workplace loneliness is linked to psychological distress, reduced well-being, and diminished workplace social support.

**What this study adds**
 

- This study presents the development and validation of 2 concise workplace loneliness measures: the SLAW-3 and the SLAW-1.
- The SLAW-3 offers a robust, reliable, and unidimensional measure, whereas the SLAW-1 provides a valid single-item alternative, making it suitable for large-scale surveys or rapid assessments.

**How this study might affect research, practice, or policy**
 

- Researchers can benefit from the efficient tools proposed in this study to measure workplace loneliness, thereby enhancing comparability across studies and promoting broader adoption of loneliness assessments.
- Organizations and practitioners can leverage these measures to identify employees at risk of loneliness and to implement targeted interventions.
- Policy makers may consider these validated scales as part of workplace health and well-being programs, fostering more supportive work environments.

## 1. Introduction

Loneliness is a subjective emotional state arising from a gap between desired and actual social relationships, and it is often linked to poor health and well-being.^1,2^ Workplace loneliness is a domain-specific loneliness that emerges in social work environments where social interactions are influenced by organizational culture, job roles, and hierarchical structures.^3^ Workplace loneliness has been linked to adverse health and well-being of workers. A growing body of research highlights the detrimental consequences of workplace loneliness, linking it to psychological distress,^4^ burnout, and reduced work engagement and job satisfaction.^5^ It also weakens collaboration, knowledge sharing, and team-based activities, reducing morale and productivity,^6,7^ as well as leading to high job turnover.^8^ The prevalence of workplace loneliness was reportedly high (10% in the United Kingdom and 8% in Japan),^9,10^ when it was measured using single direct questions (such as “At work, how often do you feel lonely?”),^9,10^ which were not validated.

Several scales have been developed to assess workplace loneliness. The Workplace Loneliness Scale (LAWS) is the most widely used measure to evaluate feelings of negative deprivation and lack of social companionship at work.^3^ Although the LAWS is a well-validated measure, it is lengthy (16 items) and thus may be burdensome for large-scale or repeated surveys; it also includes reverse-coded items that hinder simple reduction and provides no direct single-item indicator for rapid workplace screening.

Measuring “general” loneliness typically involves both direct and indirect methods, each with distinct strengths and limitations.^4^ Indirect methods assess loneliness by asking about perceived social connection (eg, “I have someone to talk to when I need help”), trying to reduce response bias.^1,11^ Many scales have been developed and validated for the indirect method, such as the Revised UCLA Loneliness Scale (R-UCLA).^11–13^ A shorter scale was also developed. For instance, the Three-Item Loneliness Scale^14^ was developed using items drawn from the full version of R-UCLA.^11^ By contrast, a direct item explicitly asking about loneliness (eg, “I feel lonely”) has been also used, offering high face and construct validity, although it may be susceptible to social desirability bias.^4^

A potential approach to develop a shorter scale of workplace loneliness may be to follow the methodology used for measuring workplace loneliness, rather than shortening the LAWS. An earlier attempt already modified a 4-item R-UCLA scale to develop a measure of workplace loneliness by adding “at work” to each of the items.^15^ However, the scale showed only moderate reliability, possibly due to the use of both positively and negatively worded items.^15,16^ It may be a promising approach to develop a negatively worded 3-item scale and/or a single direct item scale as new short measures of workplace loneliness.

The present study aimed to developed 2 brief workplace loneliness measures and test reliability and validity of these scales in a population of Japanese employees. Specifically, we developed the Three-Item Scale for Loneliness at Work (SLAW-3) and the Single-Item Scale for Loneliness at Work (SLAW-1), based on the Three-Item Scale for Loneliness^14^ and a direct item scale.^4,17^ The SLAW-3 indirectly assesses loneliness at work through perceived social connection, whereas the SLAW-1 directly assesses workplace loneliness.

## 2. Methods

### 2.1. Participants and procedures

Data were collected through online surveys from full-time workers aged 22-63 years, recruited via Rakuten Insight, Inc. To ensure demographic balance, participants were stratified by gender and age groups (22-29, 30-39, 40-49, and 50-63 years). This survey was part of a multi-wave longitudinal project investigating how work style changes during the COVID-19 pandemic impacted Japanese employees' well-being (for additional details, see https://hp3.jp/project/study-on-covid-19-and-worker-well-being).

The project began in June 2020 with 1600 participants in the first wave (T1), and an additional 800 participants were recruited at the eighth wave (T8), resulting in a panel of 2400 respondents by the ninth wave (T9). Surveys were conducted approximately every 6 months from T7 onward. The present study used data from the 12th wave (T12), conducted in December 2024, because this was the first wave in which the newly developed workplace loneliness scales (SLAW-3 and SLAW-1) were included to examine their reliability and validity. At T12, invitations were sent to all 2400 panel members. Of these, 1380 completed the T12 survey before eligibility screening. In this article, “non-respondents” refers to wave-level nonresponse at T12 (ie, invitees who did not complete the T12 survey), not permanent cohort dropout. Eligible participants were full-time employees (regular employees or civil servants). We excluded nonregular (eg, part-time, temporary, contract) workers and those on childcare leave at T12. The analytic sample included 1228 individuals who were either regular employees or civil servants.

### 2.2. Scale development

#### 2.2.1. Three-item Scale for Loneliness at Work (SLAW-3)

We developed the SLAW-3 based on the Three-Item Loneliness Scale^14^ and its Japanese version.^18^ When developing the SLAW-3, we addressed 2 psychometric risks relevant to brief adaptations of R-UCLA items: the possibility of only moderate internal consistency and the method effects that can arise from mixing positively and negatively worded items.^12,16^ Accordingly, all 3 SLAW-3 items were written with uniform polarity and parallel phrasing, while retaining the original content domains (lack of companionship, belonging/alienation, and isolation).

With permission from these original authors, we modified each item by adding the phrase “at work” to capture loneliness experienced specifically in work contexts. The scale assessed 3 core aspects of workplace loneliness: perceived lack of companionship, feelings of alienation, and a sense of social isolation within the workplace. Participants responded on a 5-point Likert scale from 1 “Never” to 5 “Often/Almost always.” The 3 items were: “At work, do you feel a lack of companionship?” “At work, do you feel alienated?” and “At work, do you feel isolated from others?” This response format was based on the British Red Cross survey to ensure consistency with established loneliness measures.^9^ Alternative response formats were initially considered during the item development phase, but a 5-point scale was ultimately adopted for both the SLAW-3 and SLAW-1 to maintain consistency and comparability across both measures. Although no separate pilot was conducted among general workers, the wording was reviewed by researchers in occupational mental health to ensure its appropriateness for working populations.

#### 2.2.2. Single-item Scale for Loneliness at Work (SLAW-1)

The SLAW-1 scale was developed to assess workplace loneliness directly. It was derived from the general loneliness item in the Japanese Cabinet Secretariat’s “Survey on social connections,” with the phrase “at work” included at the beginning of the question for contextualization.^17^ The final item used in this study was: “At work, how often do you feel lonely?” Responses were recorded on a 5-point Likert scale—identical to the format used in the Japanese Cabinet Secretariat survey—from 1 “Never” to 5 “Often/Almost always.”

### 2.3. Measurements

The participants were asked to fill in the SLAW-3 and SLAW-1.

#### 2.3.1. Other measures for scale validation

##### 2.3.1.1. Workplace Loneliness Scale (LAWS)

Workplace loneliness was measured using the 16-item Loneliness at Work Scale (LAWS), which assesses perceived emotional and social disconnection in the workplace.^3^ The scale comprises 2 subscales: emotional deprivation (9 items) and social companionship (7 items). All social companionship items and selected emotional deprivation items were reverse-coded prior to analysis. Participants rated each item on a 7-point Likert scale ranging from 1 “Strongly disagree” to 7 “Strongly agree.” Higher scores indicated stronger feelings of workplace loneliness. In the present study, Cronbach α coefficients were .94 for emotional deprivation and .94 for social companionship. A Japanese version of the scale has been developed and validated.^19^

##### 2.3.1.2. Workplace Isolation Scale (WIS)

Workplace isolation was measured using the 10-item Workplace Isolation Scale (WIS), which evaluates perceived disconnection in professional contexts.^20^ The scale comprises 2 subscales: colleague isolation (5 items) and company isolation (5 items). All items were positively worded and reverse-coded prior to analysis. Participants rated each item on a 7-point Likert scale ranging from 1 “Strongly disagree” to 7 “Strongly agree.” Higher scores indicated stronger feelings of workplace isolation. In the present study, Cronbach α coefficients were .94 for colleague isolation and .92 for company isolation. A Japanese version of the scale has been developed and validated.^21^ Whereas workplace loneliness reflects the subjective emotional experience of lacking meaningful social connection at work, workplace isolation refers to an objective lack of communication or social interaction within the organization. Both concepts are related but distinct; examining correlations with both measures allowed us to evaluate whether the new scales captured not only emotional but also structural aspects of workplace disconnection.

##### 2.3.1.3. General Loneliness Measure

General loneliness was assessed using a single-item measure developed by the Japanese Cabinet Secretariat.^17^ Participants were asked, “How often do you feel lonely?” Responses were recorded on a 5-point Likert scale ranging from 1 “Never” to 5 “Frequently/Always.” Higher scores indicated greater feelings of loneliness. This measure has been used in national surveys to monitor levels of social and emotional isolation.

##### 2.3.1.4. Psychological distress: Kessler 6 (K6) scale

Psychological distress was assessed using the Kessler 6 (K6) scale, a 6-item instrument developed to screen for nonspecific psychological distress.^22^ Each item measures how frequently participants experienced symptoms such as “feeling so sad that nothing could cheer you up” during the past 30 days. Participants responded using a 5-point Likert scale ranging from 0 “None of the time” to 4 “All of the time.” Each item was scored from 0 to 4, resulting in a total score range of 0 to 24. Higher scores indicated greater psychological distress. In the present study, the Cronbach α coefficient was .95. A Japanese version of the K6 scale has been validated and widely used for screening mental disorders.^23^

##### 2.3.1.5. WHO-5 Well-Being Index

Psychological well-being was assessed using the WHO-5 Well-Being Index, a 5-item self-report measure developed by the World Health Organization.^24^ A validated Japanese version of the WHO-5 was used in this study.^25^ All items are positively worded and assess how frequently respondents experienced positive mental states during the past 2 weeks. Participants responded using a 6-point Likert scale ranging from 0 “At no time” to 5 “All of the time.” The total score ranges from 0 to 25, with higher scores indicating greater well-being. In the present study, the Cronbach α coefficient was .94.

##### 2.3.1.6. Self-rated health: single-item measure

Self-rated health was assessed using a single-item measure that asked participants to evaluate their overall health status: “How would you rate your current health condition?” Responses were recorded on a 5-point scale, ranging from 1 “Poor” to 5 “Excellent.” Higher scores indicated better perceived health. Previous research shows that overall self-rated health can be captured adequately with a single-item measure.^26^

##### 2.3.1.7. Subjective happiness: single-item measure

Subjective happiness was assessed using a single-item question: “Overall, how happy do you feel in your life?” Responses were recorded on a 10-point scale, from 1 “Not happy at all” to 10 “Extremely happy.” Higher scores indicated greater levels of subjective happiness. Single-item happiness scales have shown acceptable reliability and construct validity.^27^

##### 2.3.1.8. Brief Job Stress Questionnaire (BJSQ)

Workplace social support was assessed using a 6-item scale derived from the Brief Job Stress Questionnaire (BJSQ), developed to measure perceived support from supervisors and coworkers in the workplace. The scale comprises 2 subscales: supervisor support (3 items) and coworker support (3 items).^28,29^ Participants rated how much support they received using a 4-point Likert scale ranging from 1 “Not at all” to 4 “Very much.” Higher scores indicated greater perceived social support. In the present study, the Cronbach α coefficients were .88 for supervisor support and .88 for coworker support.

### 2.4. Demographic variables

Demographic characteristics such as age, gender, average working hours per week, marital status, educational attainment, employment status, job rank, industry type, and work schedule were included in the questionnaire as potential confounding factors. Demographic and occupational characteristics (eg, age, marital status, job rank) were collected and reported to characterize the sample and aid interpretation/generalizability. Prior studies have found higher workplace loneliness among younger employees, unmarried individuals, and those in lower occupational positions.^10^ These variables were not entered as covariates in the validation analyses.

### 2.5. Statistical analysis

The reliability of the SLAW-3 was assessed using Cronbach α to evaluate internal consistency. Additionally, a principal component analysis (PCA) was conducted to examine dimensionality, with principal component loadings, contribution ratios, and Cronbach α coefficients calculated to evaluate internal consistency and factorial structure.

Construct validity of the SLAW-3 and SLAW-1 was assessed through polychoric correlation analysis with related measures. SLAW-3 and SLAW-1 were expected to correlate positively with existing workplace loneliness measures (Workplace Loneliness Scale and Workplace Isolation Scale) and psychological distress (K6), and negatively with well-being (WHO-5) and workplace social support (BJSQ). Self-rated health and subjective happiness were also included as indicators of well-being.

### 2.6. Ethics

This study was approved by 2 institutional ethics review boards (approval numbers: 20E0004, 336, 404, 420, 433, 455, 482, and 549). It was registered and conducted as a clinical trial (UMIN study ID: UMIN000040683), titled “Longitudinal study on work style change, health, and well-being owing to pandemic of novel coronavirus infection (COVID-19).” The UMIN registration originally specified an age range of 20-60 years, reflecting the participant composition at the project’s inception (T1) in 2020. The present analysis used data from the 12th wave (T12), where the age range (22-63 years) naturally widened due to the aging of the longitudinal cohort over the 4-year study period. This is a common and expected occurrence in longitudinal research and does not represent a deviation from the registered protocol. The analysis focused on data collected in December 2024, when the acute phase of the COVID-19 pandemic had subsided; thus, pandemic-related effects were not directly analyzed in this study.

## 3. Results

### 3.1. Participant characteristics

Table 1 shows the participant characteristics. Participants (*n* = 1228) were 55% men. Most worked a fixed daytime schedule (73%) and 40-44 hours per week (40%), while 6% worked more than 60 hours per week. About two-fifths held managerial posts, with clerical and professional/technical roles most common; manufacturing (19%) and health care/welfare (11%) were the leading industries. A majority were university graduates (57%), and over half were married (53%).

**Table 1:** Participant characteristics (*n* = 1228).

**Variable [possible range]**	***n* (%)**
**Mean (SD) age, y [22-63]**	44.80 (10.29)
**Gender**	
**Male**	670 (54.6)
**Female**	558 (45.4)
**Work schedule**	
** Fixed daytime schedule**	893 (72.7)
** Shortened working hours**	21 (1.8)
** Flextime system**	141 (11.5)
** Deemed working hours system**	41 (3.3)
** Shift work including night shifts**	113 (9.2)
** Shift work excluding night shifts**	19 (1.5)
**Weekly working hours**	
** 40 h or less**	246 (20.1)
** 40-44 h**	496 (40.4)
** 45-49 h**	191 (15.6)
** 50-54 h**	199 (16.2)
** 55-59 h**	19 (1.5)
** 60-64 h**	42 (3.4)
** 65-69 h**	6 (0.5)
** 70-74 h**	15 (1.2)
** 75 h or more**	14 (1.1)
**Occupation** ^**a**^	
** Managerial**	134 (10.9)
** Professional/technical**	268 (21.8)
** Clerical/administrative**	395 (32.3)
** Sales/service**	175 (14.2)
** Others**	256 (20.9)
**Industry** ^**a**^	
** Manufacturing/construction**	332 (27.0)
** Information/finance/services**	368 (30.0)
** Education/health care/welfare**	221 (18.0)
** Public sector**	96 (7.8)
** Others/unclassifiable**	211 (17.2)
**Job rank**	
** Manager**	488 (39.7)
** Nonmanager**	740 (60.3)
**Educational attainment**	
** Junior high school**	8 (0.6)
** High school**	263 (21.4)
** Vocational school**	152 (12.4)
** Junior college/technical college**	103 (8.4)
** University**	604 (49.2)
** Graduate school**	93 (7.6)
** Other**	5 (0.4)
**Marital status**	
** Married**	648 (52.8)
** Single/not married**	580 (47.2)

Percentages are of the total sample (*n* = 1228).

^a^To improve clarity, occupation and industry categories with small sample sizes were merged into broader groups.

### 3.2. Scale for Loneliness at Work (SLAW-3)

Of the 1380 respondents in T12, we excluded 152 participants based on predefined criteria: 136 were not full-time employees (eg, part-time, temporary, or contract workers) and 16 were on childcare leave. The final analytic sample comprised 1228 participants (670 men, 558 women; mean age = 44.80, SD = 10.29).

#### 3.2.1. Internal consistency

The internal consistency of the SLAW-3 was evaluated using Cronbach α, which demonstrated a high reliability of .91.

#### 3.2.2. Factor structure and principal component analysis

Principal component analysis was conducted to examine the structural validity of the SLAW-3. The analysis revealed that the first principal component accounted for 85.2% of the total variance, confirming the scale’s unidimensionality.

Factor loadings for each respective item in the first principal component were as follows: “At work, do you feel that you lack companionship?” with a factor loading of 0.88; “At work, do you feel alienated?” with a factor loading of 0.94; and “At work, do you feel isolated from others?” with a factor loading of 0.94. These high factor loadings indicate that all 3 items effectively measure the single underlying construct of workplace loneliness. The results are presented in Table 2.

**Table 2:** Principal component analysis results for the SLAW-3.

**Item**	**Mean**	**SD**	**Skewness**	**Kurtosis**	**Correlation**			**Factor loading**
					**1**	**2**	**3**	
**1. At work, do you feel a lack of companionship?**	2.80	1.17	0.27	−0.70	—			0.88
**2. At work, do you feel alienated?**	2.48	1.09	0.50	−0.24	0.73	—		0.94
**3. At work, do you feel isolated from others?**	2.55	1.13	0.43	−0.47	0.73	0.88	—	0.94
**Three-Item Workplace Loneliness Scale (Total)**	7.82	3.13	0.36	−0.31	0.89	0.94	0.94	—

Abbreviation: SLAW-3, 3-item Scale for Loneliness At Work.

#### 3.2.3. Construct validity: correlation with other scales

The SLAW-3 demonstrated significant positive correlations with established measures of workplace disconnection, confirming its construct validity. It correlated positively with the Loneliness at Work Scale (*r* = 0.64, for lack of social companionship; *r* = 0.46, for emotional deprivation) and the Workplace Isolation Scale (*r* = 0.50, for colleague isolation; *r* = 0.51, for company isolation).

Additionally, workplace loneliness as measured by the SLAW-3 exhibited a moderate correlation with general loneliness (*r* = 0.75), indicating shared characteristics while maintaining conceptual distinctiveness.

Furthermore, SLAW-3 scores were moderately to strongly correlated with psychological distress (K6, *r* = 0.57) and negatively correlated with well-being (WHO-5, *r* = −0.34), self-rated health (*r* = −0.27), and subjective happiness (*r* = −0.33), indicating consistent associations with adverse psychological and health-related outcomes.

Moreover, SLAW-3 scores were negatively correlated with supervisor support (*r* = −0.35) and coworker support (*r* = −0.40), further demonstrating that lower workplace social support is associated with greater workplace loneliness.

### 3.3. Single-item Scale for Loneliness at Work (SLAW-1)

The same 1228 participants responded to the SLAW-1, which was examined alongside the SLAW-3. The mean, SD, skewness, and kurtosis for the SLAW-1 were 2.62, 1.13, 0.39, and −0.53, respectively (Table 3).

**Table 3:** Descriptive statistics for the single-item SLAW-1.

**Item**	**Mean**	**SD**	**Skewness**	**Kurtosis**
**At work, how often do you feel lonely?**	2.62	1.13	0.39	−0.53

#### 3.3.1. Validity and correlation analysis

To evaluate the validity of the SLAW-1, analyses were conducted utilizing a 5-point Likert scale. The SLAW-1 exhibited significant positive correlations with the SLAW-3 (*r* = 0.73), the Loneliness at Work Scale (*r* = 0.58, for social companionship; *r* = 0.46, for emotional deprivation) and the Workplace Isolation Scale (*r* = 0.50, for colleague isolation; *r* = 0.49, for company isolation). Furthermore, a strong positive correlation with general loneliness (*r* = 0.75) confirmed its validity as a measure of workplace loneliness.

Additionally, the SLAW-1 was moderately correlated with psychological distress (K6, *r* = 0.51) and negatively correlated with well-being (WHO-5, *r* = −0.38), self-rated health (*r* = −0.40), and subjective happiness (*r* = −0.46). Similar to the SLAW-3, workplace loneliness measured by the SLAW-1 negatively correlated with supervisor support (*r* = −0.34) and coworker support (*r* = −0.39), suggesting that lower workplace social support is associated with greater loneliness. These correlations are summarized in Table 4.

**Table 4:** Construct validity: polychoric correlations between SLAW-3/SLAW-1 and criterion measures.

**Criterion measure**	**SLAW-3**	**SLAW-1**
**Social companionship (LAWS)**	0.64	0.58
**Emotional deprivation (LAWS)**	0.46	0.46
**Colleague isolation (WIS)**	0.50	0.50
**Company isolation (WIS)**	0.51	0.49
**General loneliness measure**	0.75	0.75
**Psychological distress (K6)**	0.57	0.51
**WHO-5 Well-Being Index**	−0.34	−0.38
**Self-rated health**	−0.27	−0.40
**Subjective happiness**	−0.33	−0.46
**Supervisor support (BJSQ)**	−0.35	−0.34
**Coworker support (BJSQ)**	−0.40	−0.39

Abbreviations: BJSQ, Brief Job Stress Questionnaire; K6, Kessler 6; LAWS, Workplace Loneliness Scale; SLAW-1, single-item Scale for Loneliness At Work; SLAW-3, 3-item Scale for Loneliness At Work; WIS, Workplace Isolation Scale.

All coefficients are polychoric *r* values (*n* = 1228), *P* < .001 for all. Higher scores indicate greater levels of each construct, except WHO-5, self-rated health, and subjective happiness for which higher scores represent better states; therefore, negative *r* denotes greater loneliness associated with poorer well-being/support.

## 4. Discussion

This study developed and validated the SLAW-3 and SLAW-1 as brief measures of workplace loneliness. Both scales demonstrated strong reliability and validity.

The SLAW-3 exhibited high internal consistency and showed significant positive correlations with existing measures of workplace disconnection, including the Loneliness at Work Scale and the Workplace Isolation Scale.^3,20^ The internal consistency of the SLAW-3 (Cronbach α = .91) was substantially higher than that reported for the previous 4-item workplace loneliness adaptation (α = .68),^15^ suggesting improved reliability through consistent item phrasing. This finding supports the convergent validity of the scale. Furthermore, the SLAW-3 was positively associated with psychological distress (K6) and negatively correlated with psychological well-being (WHO-5), self-rated health, and subjective happiness. It was also negatively associated with perceived social support from supervisors and coworkers, suggesting that lower perceived social support is consistently correlated with higher workplace loneliness. These findings align with prior research linking workplace loneliness to adverse psychological and health-related outcomes.^2,5^

The PCA results confirmed the unidimensionality of the scale, with the first principal component accounting for 85.2% of the variance. The high factor loadings across all 3 items (ranging from 0.88 to 0.94) further support its structural validity. Given these strong psychometric properties, the SLAW-3 is a reliable tool for assessing workplace loneliness in both research and applied settings.

The SLAW-1 was developed as a concise, direct measure of workplace loneliness. It demonstrated significant correlations with the SLAW-3 and other workplace loneliness measures. Furthermore, it exhibited strong associations with psychological distress, well-being, and workplace support, reinforcing its applicability as a valid indicator of workplace loneliness. These correlations indicate that the SLAW-1 possesses adequate construct validity, despite its single-item format. Both the SLAW-3 and SLAW-1 showed strong correlations with the General Loneliness Measure, which also supports the validity of the scales, because workplace loneliness is theoretically considered as a subset of general loneliness. It is slightly puzzling that these correlation coefficients were greater than those of SLAW-3 and SLAW-1 with LAWS, raising a question about the discriminant validity of the new scales. This finding is partly attributable to a same-method bias: SLAW-3, SLAW-1, and the General Loneliness Measure use negatively worded questions and frequency-based response options; LAWS uses both negatively and positively worded questions and agreement-based response options. However, it should be further tested if the SLAW-3 and SLAW-1 specifically measure workplace loneliness, separately from general loneliness.

Although the SLAW-1 effectively measures key aspects of workplace loneliness, its single-item format may limit its ability to assess the full spectrum of workplace loneliness experiences. However, given its simplicity and ease of administration, the SLAW-1 serves as a practical, quick, screening tool for workplace mental health screenings.

One key advantage of the SLAW-1 is its practicality for large-scale workplace assessments, particularly in workplace settings where time constraints may limit the feasibility of multi-item scales. Whereas traditional workplace loneliness measures require multiple items, the SLAW-1 enables quick assessments, making it particularly valuable for workplace mental health screening and longitudinal studies requiring repeated measurements. However, it should be utilized as a complementary measure rather than a substitute for more comprehensive tools like the SLAW-3.

Since both scales were administered to the same sample of 1228 participants, direct comparisons were possible. The substantial correlation between the 2 scales (*r* = 0.73) suggests that although the SLAW-1 captures core aspects of workplace loneliness, it does not fully replace the detailed insights provided by the SLAW-3. Given their complementary strengths, the 2 scales can be utilized together to enhance workplace mental health assessments, with the SLAW-3 offering detailed evaluations and the SLAW-1 enabling rapid screenings.

Future research should examine the predictive validity of these scales in relation to workplace outcomes such as job satisfaction, employee turnover, and overall well-being. Longitudinal studies would be particularly valuable in understanding the long-term effects of workplace loneliness and the potential protective role of workplace social support. As workplace loneliness has been linked to negative outcomes in mental health, job performance, and productivity,^1,3^ having brief yet psychometrically sound measurement tools is essential. The SLAW-3 and SLAW-1 provide organizations with tools to identify at-risk employees and evaluate the effectiveness of targeted interventions, supporting both research and practical applications. At the same time, when applying these scales in workplace contexts, ethical concerns may arise depending on how they are administered and used. Practically, these tools should be implemented under clear data protection and information governance safeguards—purpose and lawful basis (with consent where appropriate), minimal collection, secure access-controlled storage, no unauthorized third-party disclosure, deidentified/aggregated reporting, limited retention, and procedures for access and correction—with voluntary participation and without use of results for individual personnel decisions.

This study had some limitations. First, its cross-sectional design prevents causal inferences regarding the relationship between workplace loneliness and psychological outcomes. Second, the study sample consisted only of full-time employees in Japan, which limits the generalizability of the findings to other groups, such as nonregular employees, freelance workers, or those in different cultural contexts. Future research should examine the applicability and validity of the SLAW-3 and SLAW-1 across diverse occupational settings and employment statuses to confirm their broader utility. Third, although this study demonstrated high internal consistency for both scales, we did not conduct a test–retest analysis across 2 stable time points. Future research should examine the temporal stability of the SLAW-3 and SLAW-1 to further confirm their reliability. Known-groups validity was not examined in the present study. Future research should evaluate it by comparing SLAW scores across theoretically relevant subgroups (eg, age, marital status, job rank). Lastly, wave-level nonresponse at T12 may introduce selection bias. Therefore, generalizability should be interpreted with caution.

## 5. Conclusions

This study developed and validated 2 brief measures of workplace loneliness: the SLAW-3 and SLAW-1. The findings demonstrate that both scales exhibit strong reliability and validity, making them valuable tools for assessing workplace loneliness. The SLAW-3 provides a robust, unidimensional measure suitable for in-depth research and psychological evaluation, whereas the SLAW-1 offers a concise and practical alternative for rapid workplace mental health screenings. These scales can support both research and organizational efforts to identify and address workplace loneliness, ultimately contributing to improved employee well-being and workplace interventions.

## Data Availability

The data supporting this study’s findings are available from the corresponding author upon reasonable request.
